# Seasonal Population Dynamics of Mosquitoes in Taipei, Taiwan

**DOI:** 10.3390/insects17060592

**Published:** 2026-06-05

**Authors:** Da-Gang Huang, Hsin-Chieh Tang, Chi-Wei Tsai

**Affiliations:** 1 Department of Entomology, National Taiwan University, Taipei 106, Taiwan; r11632003@ntu.edu.tw; 2 Conservation and Research Center, Taipei Zoo, Taipei 116, Taiwan; tgx02@zoo.gov.tw; 3 Department of Veterinary Medicine, National Taiwan University, Taipei 106, Taiwan

**Keywords:** *Aedes*, *Culex*, mosquito, population dynamics, seasonal dynamics, vector

## Abstract

Mosquito-borne diseases pose a significant public health concern globally; however, data on mosquito population dynamics in Taipei, Taiwan are limited and outdated. Updated information on species composition and seasonal abundance is crucial for enhancing vector surveillance and informing effective control strategies. In this study, to investigate the seasonal dynamics of mosquito populations in Taipei, Taiwan, adult females were collected biweekly from June 2023 to May 2025 using CDC light traps baited with ultraviolet light and dry ice. Species identification was performed based on morphological characteristics, and morphologically challenging *Culex* mosquito species were further confirmed using cytochrome c oxidase I barcoding. Mosquito surveillance from June 2023 to May 2025 yielded 1926 females representing 31 species. Of these, *Culex quinquefasciatus*, *Culex pipiens molestus*, *Aedes albopictus*, and *Culex tritaeniorhynchus* accounted for over 90% of all specimens. These dominant species exhibited distinct seasonal patterns: *Cx. quinquefasciatus* occurred year-round, *Cx. pipiens molestus* predominated during winter and spring, while *Ae. albopictus* and *Cx. tritaeniorhynchus* populations peaked in summer. Furthermore, spatial heterogeneity in both mosquito abundance and species composition was noted among the study sites. Monitoring the composition and seasonal dynamics of mosquito species is essential for understanding the epidemiology of mosquito-borne pathogens. These insights can inform more effective and targeted vector control strategies for reducing disease transmission. Such ecological insights can also support One Health approaches by linking human, animal, and environmental factors that influence the transmission of mosquito-borne diseases.

## Simple Summary

Beyond being a persistent nuisance, mosquitoes serve as primary vectors for pathogens that pose a global threat to human health. In Taipei, Taiwan, however, comprehensive data regarding mosquito population dynamics has remained outdated for decades. To address this gap, we conducted a two-year survey from June 2023 to May 2025, employing biweekly trapping of mosquitoes using ultraviolet light and dry ice at three locations. Our surveillance yielded 1926 female mosquito specimens across 31 species. Notably, four species (*Culex quinquefasciatus*, *Culex pipiens molestus*, *Aedes albopictus*, and *Culex tritaeniorhynchus*) accounted for over 90% of the total collection. These dominant species exhibited distinct seasonal patterns: *Cx. quinquefasciatus* was present year-round, *Cx. pipiens molestus* thrived during the cooler winter and spring, while *Ae. albopictus* and *Cx. tritaeniorhynchus* populations peaked in summer. Given the significant spatial and temporal variations in mosquito composition, continuous monitoring of these dynamics is fundamental to effective disease management. By leveraging these ecological insights, public health authorities can optimize resource allocation and execute precision-targeted interventions. Furthermore, integrating this ecological data into a One Health framework is essential for disrupting the cross-species transmission of pathogens.

## 1. Introduction

Mosquitoes (Diptera: Culicidae) are among the most medically important arthropods. They act as vectors for various pathogens that cause substantial morbidity and mortality worldwide [1,2]. Key genera of public health concern include *Anopheles*, which is associated with malaria; *Culex*, which transmits lymphatic filariae and encephalitis viruses; and *Aedes*, which transmits dengue virus (DENV), Zika virus (ZIKV), and chikungunya virus (CHIKV) [3]. Collectively, mosquito-borne diseases pose a major threat to public health globally, resulting in more than one billion diagnosed cases and over one million deaths worldwide annually [2]. These diseases also impose a heavy burden on healthcare systems and economies, hindering agricultural productivity, urban development, and overall socioeconomic progress [4].

In recent decades, the incidence and geographic range of mosquito-borne diseases have significantly increased, with many pathogens emerging in new areas or reemerging where transmission had previously been eradicated, e.g., DENV and yellow fever virus [5]. These changes present escalating public health challenges. Previous studies have indicated that various factors, such as global environmental change, climate variability, land use transformation (including agriculture, deforestation, and urbanization), and socioeconomic development, collectively contribute to the emergence and spread of mosquito-borne diseases [5,6,7,8,9]. Moreover, the seasonal patterns of mosquito abundance are crucial for assessing the infection risk of these diseases [10,11]. Therefore, assessment of the distribution and abundance of mosquitoes in specific geographic areas is essential for advancing our understanding of the eco-epidemiology of mosquito-borne diseases.

A total of 16 genera and 132 mosquito species belonging to the family Culicidae have been recorded and described in Taiwan [12]. Among these, several species are of major medical and veterinary importance, including *Aedes aegypti*, *Aedes albopictus*, *Culex quinquefasciatus*, *Culex tritaeniorhynchus*, *Culex fuscocephala*, and *Anopheles minimus* [13]. However, research on mosquito population dynamics in Taiwan is limited and largely outdated. Prior studies have mainly focused on population fluctuations of individual vector species associated with specific mosquito-borne diseases, e.g., *Cx. tritaeniorhynchus*, *Ae. aegypti*, and *Cx. quinquefasciatus* [14,15]. In the case of dengue and malaria vectors, some studies have examined the seasonal abundance of larval populations [16,17,18,19]. To date, only one systematic survey has documented the seasonal succession of 11 mosquito species in Taipei from 1960 to 1963 [20]. However, since then, substantial climatic and environmental changes, along with the extensive use of insecticides and disinfectants, may have significantly impacted mosquito breeding and survival in the region. Therefore, updated investigations of mosquito species composition and seasonal population dynamics are urgently required to capture recent ecological shifts and strengthen contemporary vector surveillance and control strategies.

The objectives of this study were to investigate the species composition, seasonal abundance, and spatial variability of mosquitoes in Taipei, Taiwan, to address the lack of updated and comprehensive data on mosquito population dynamics in subtropical cities. Beyond ecological characterization, our findings provide a pragmatic framework for vector management. Specifically, by identifying species-specific seasonal peaks, this study offers an empirical basis for synchronizing control interventions with periods of maximum vector activity. Furthermore, the observed spatial variability supports the development of site-specific, rather than uniform, surveillance protocols. Collectively, these results facilitate the transition from reactive to proactive, evidence-based mosquito management strategies in Taipei and comparable urban subtropical regions.

## 2. Materials and Methods

### 2.1. Study Sites

**Figure 1:**
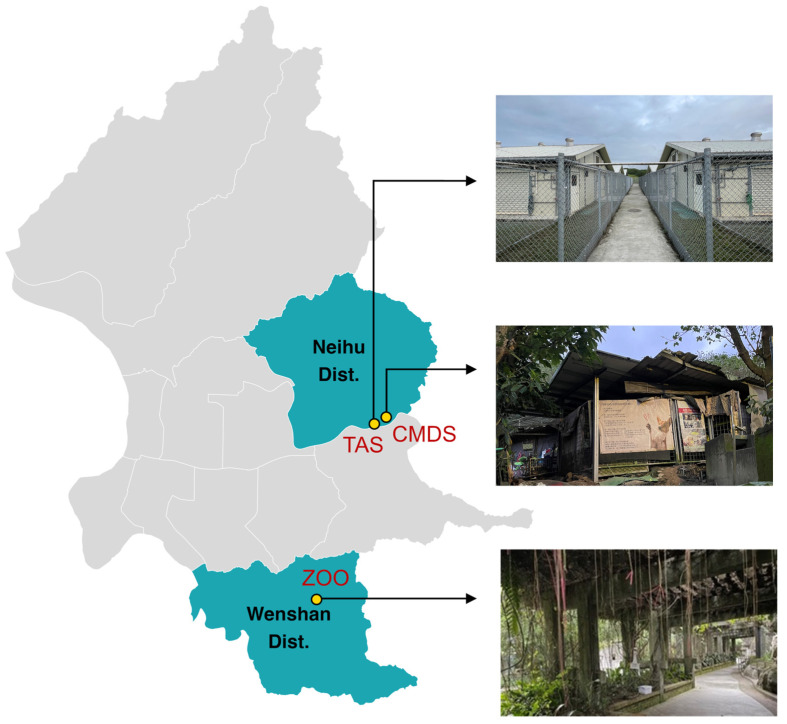
Three study sites in Taipei, Taiwan. CMDS: Cho Mama Dog Shelter; TAS: Taipei Animal Shelter; ZOO: Taipei Zoo.

The study was conducted in Taipei, a city in Taiwan characterized by a subtropical climate. According to records from the Climate Observation Data Inquiry System of the Central Weather Administration, Taiwan, the average monthly temperature in Taipei during 2023–2025 ranged from 15.5 °C to 30.7 °C, with the annual precipitation exceeding 2000 mm and relative humidity (RH) varying between 65% and 85%. Meteorological parameters, including monthly temperature, precipitation, and RH during the study period, are summarized in Table S1. Mosquitoes were collected from three locations (Figure 1): Cho Mama Dog Shelter (CMDS, 25°03′46.8″ N 121°36′28.9″ E), Taipei Animal Shelter (TAS, 25°03′38.2″ N 121°36′12.1″ E), and Taipei Zoo (ZOO, 24°59′53.0″ N 121°34′49.6″ E). CMDS and TAS are located in the Neihu District, within the Keelung River basin, whereas ZOO is located in the Wenshan District, along the Jingmei River.

CMDS is located on a rugged hillside. It originally housed 35 stray dogs in 2023. The site has limited sanitation infrastructure, which may create conditions conducive to the presence of rodents and insects. Despite these constraints, the caretaker ensures the provision of adequate food, water, and necessary supplies and regularly administers preventative medications to maintain the dogs’ health.

TAS has a capacity of up to 840 dogs and frequently operates at full occupancy. To reduce the risk of disease transmission, this shelter maintains a hygienic environment, ensures adequate ventilation, reduces overcrowding, and implements well-structured, zoned management practices. Moreover, routine preventive treatments are implemented here to maintain the dogs’ well-being.

ZOO is located adjacent to mountainous terrain and is surrounded by low-elevation broadleaf forests, with the Jingmei River nearby, creating a complex semi-natural ecosystem. Animal enclosures are designed to closely mimic natural habitats. Nevertheless, a hygienic environment is maintained through regular cleaning and maintenance. Moreover, routine preventive treatments and health screenings are implemented to reduce the risk of infectious diseases.

These sites were strategically selected to reflect varying degrees of urbanization. CMDS and TAS were chosen for their proximity to residential areas, while the ZOO site serves as a representative of a semi-natural ecosystem. Collectively, the inclusion of these contrasting locations allows for a more comprehensive characterization of mosquito diversity across the city.

### 2.2. Mosquito Collection and Identification

Mosquitoes were collected biweekly from June 2023 to May 2025 using CDC light traps (John W. Hock Company, Gainesville, FL, USA) baited with ultraviolet light and 1 kg dry ice. The traps were placed 1.5 m above ground and operated overnight (18:00–10:00). Non-mosquito insects and male mosquitoes were excluded from the study, with the latter identified based on distinct morphological features, specifically their plumose antennae and elongated maxillary palps. This gender-based sorting was conducted under a stereomicroscope to ensure that only female mosquitoes were retained. All collected females were subsequently identified to the species level under a stereomicroscope using a morphological key [12]. All samples were stored at −20 °C.

### 2.3. Molecular Identification of Mosquito Species

Specimens presenting diagnostic challenges during morphological identification were subjected to cytochrome c oxidase I (COI) barcoding [21] for taxonomic confirmation. This process primarily targeted specimens with damaged or missing key diagnostic features, such as scales on the thorax, wings, legs, and abdomen, which are essential for accurate identification. Furthermore, molecular verification was performed for *Culex* species exhibiting high morphological similarity (e.g., *Culex pallidothorax*, *Culex kyotoensis*, and *Culex sasai*), as existing taxonomic keys may not reliably differentiate these taxa. Consequently, all *Culex* species, with the exception of the common and morphologically distinct *Culex macrostylus*, *Culex mimulus*, *Culex pipiens molestus*, *Cx. quinquefasciatus*, *Culex sitiens*, and *Cx. tritaeniorhynchus*, were further validated using COI barcoding to ensure the highest level of taxonomic precision.

Mosquito DNA was extracted using the gSYNC DNA Extraction Kit (Geneaid, New Taipei, Taiwan). PCR was performed using a 10-μL reaction mixture comprising 5 µL of 2× Taq PCR Mix-RED (Bioman, New Taipei, Taiwan), 0.3 µL of each primer (10 µM; F: 5′-GGATTTGGAAATTGATTAGTTCCTT-3′; R: 5′-AAAAATTTTAATTCCAGTTGGAAC-AGC-3′) [21], 1 µL of template DNA, and 3.4 µL of double-distilled water (ddH_2_O). DNA extracted from laboratory-reared *Ae. albopictus* served as a positive control, while ddH_2_O served as a negative control in each PCR run. The thermocycling program comprised an initial denaturation step at 95 °C for 5 min; five cycles at 94 °C for 30 s, 45 °C for 30 s, and 72 °C for 1 min; 35 cycles at 94 °C for 30 s, 51 °C for 30 s, and 72 °C for 1 min; and a final extension step at 72 °C for 10 min. Amplicons (735 bp) were visualized by electrophoresis on 1% agarose gels, purified using the GenepHlow PCR Cleanup Kit (Geneaid), and subjected to Sanger sequencing. The resulting sequences were searched against the GenBank database using BLASTn 2.17.0 to identify the closest matches.

### 2.4. Statistical Analyses

To evaluate the influence of meteorological variables (temperature, RH, and rainfall) on the abundance of the four most dominant mosquito species, Generalized Linear Mixed Models (GLMMs) were employed using a negative binomial distribution and a log-link function. This model was selected to account for the overdispersion inherent in mosquito count data. The GLMM included species, site, temperature, RH, and rainfall as fixed effects. The sampling date was included as a random effect. To examine whether species-specific abundances responded differently to meteorological variables, interaction terms between species and each meteorological factor (Species × Temperature, Species × RH, and Species × Rainfall) were included. All continuous meteorological variables were Z-score standardized prior to analysis to facilitate a direct comparison of their relative importance. Statistical significance was defined at α = 0.05.

To assess mosquito community diversity across the study sites, diversity indices were calculated based on the cumulative species abundance data. Simpson index (1 − *D*) was employed to represent the probability that two individuals randomly selected from a sample belong to different species, calculated as: 1 − *D* = 1 − ∑*p_i_*^2^ [22], where *p_i_* represents the proportion of individuals belonging to the *i*th species relative to the total number of individuals. Values for the Simpson index range between 0 and 1, with larger values representing greater diversity. Additionally, Shannon’s diversity index (*H*′) was used to measure species richness and evenness, calculated as: *H*′ = −∑*p_i_* ln (*p_i_*) [22]. Larger values represent greater diversity.

## 3. Results

### 3.1. Barcoding Species Identification

To ensure taxonomic accuracy, specimens with damaged or missing key diagnostic features, as well as *Culex* species exhibiting high morphological similarity, were subjected to COI barcoding. A total of 28 *Culex* specimens were analyzed, with species identification performed using BLASTn against the GenBank database. The molecular analysis confirmed the presence of the following six species: *Culex annulus* (*n* = 2), *Culex bicornutus* (*n* = 1), *Culex bitaeniorhynchus* (*n* = 6), *Culex infantulus* (*n* = 1), *Culex nigropunctatus* (*n* = 3), and *Cx. pallidothorax* (*n* = 15).

### 3.2. Mosquito Abundance

The mosquito collection data across the three study sites are presented in Table 1. A total of 1926 specimens representing 31 species across nine genera (*Aedes*, *Anopheles*, *Armigeres*, *Culex*, *Malaya*, *Mansonia*, *Mimomyia*, *Ochlerotatus*, and *Uranotaenia*) were caught from June 2023 to May 2025. The most commonly caught species were *Cx. quinquefasciatus* (*n* = 1048; 54%), *Cx. pipiens molestus* (*n* = 369; 19%), *Ae. albopictus* (*n* = 201; 10%), and *Cx. tritaeniorhynchus* (*n* = 158; 8%); they collectively accounted for over 90% of all specimens. Other species included *Armigeres subalbatus* (*n* = 31; 1.6%), *Cx. mimulus* (*n* = 25; 1.3%), and *Cx. sitiens* (*n* = 24; 1.2%), with the remaining 24 species collectively representing 3.6% of the caught specimens.

### 3.3. Temporal Dynamics of Mosquito Abundance

**Figure 2:**
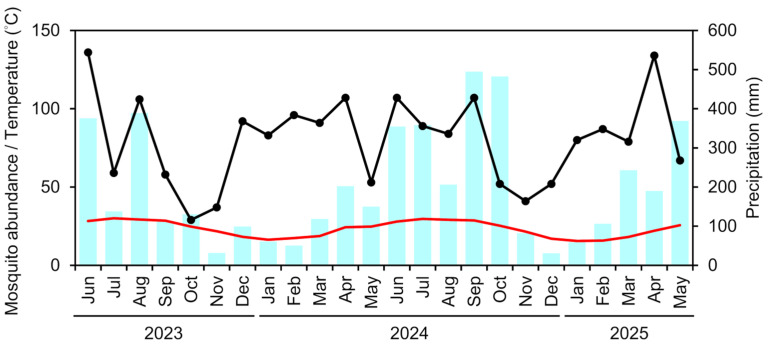
Temporal dynamics of mosquito abundance and meteorological variables in Taipei, Taiwan. The black line with markers represents total mosquito abundance. Data are overlaid with monthly average temperature (°C), indicated by the solid red line, and monthly total precipitation (mm), represented by the light blue bars.

**Figure 3:**
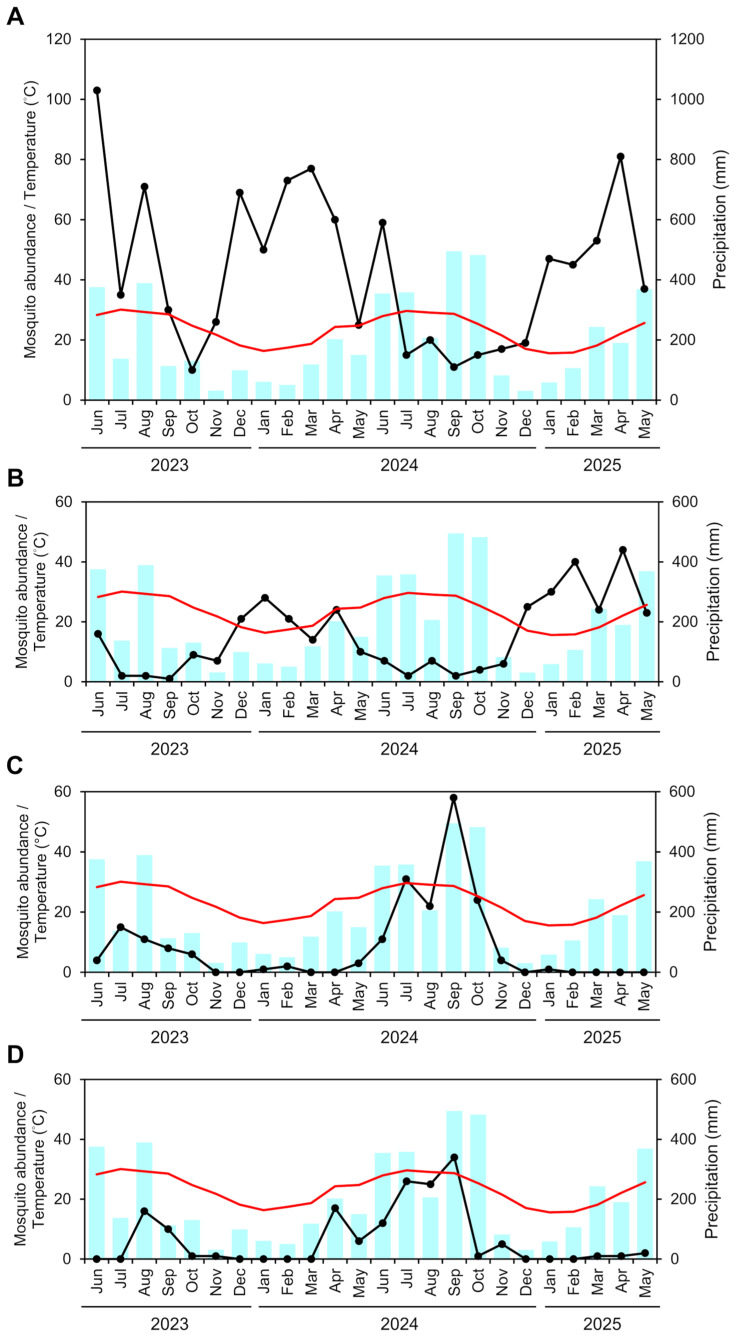
Species-specific temporal dynamics of the four most abundant mosquito species in Taipei, Taiwan. Fluctuations in the abundance of (**A**) *Culex quinquefasciatus*, (**B**) *Culex pipiens molestus*, (**C**) *Aedes albopictus*, and (**D**) *Culex tritaeniorhynchus*. In each panel, the black line with markers denotes the abundance of the respective species. The data are overlaid with monthly average temperature (°C) (solid red line) and monthly total precipitation (mm) (light blue bars).

The temporal dynamics of the mosquitoes caught across the three study sites are illustrated in Figure 2. Mosquitoes were present year-round, with population troughs recorded from September to November in 2023 and from October to December in 2024. The temporal dynamics of the four most abundant mosquito species are illustrated in Figure 3. *Culex quinquefasciatus* was the most prevalent species throughout the two-year study period; its prevalence was the lowest in September–November 2023 and July–December 2024 (Figure 3A). The population of *Cx. pipiens molestus* peaked in winter and spring (from December to April, Figure 3B). *Aedes albopictus* was primarily collected in summer (between June and October, Figure 3C), while *Cx. tritaeniorhynchus* was primarily collected between April and September, with its presence peaking in August and September (Figure 3D).

GLMMs were employed to evaluate the influence of meteorological variables on the abundance of the four most dominant mosquito species. The results identified temperature and RH as the primary drivers of abundance (temperature: *F* = 25.41, *p* < 0.001; RH: *F* = 4.02, *p* = 0.045), while the effect of rainfall was not statistically significant (*F* = 0.57, *p* = 0.452). Regarding species-specific responses, temperature was positively correlated with the abundance of *Ae. albopictus* (β = 0.457, *p* < 0.001) and *Cx. tritaeniorhynchus* (β = 0.294, *p* < 0.001). Conversely, temperature showed a significant negative correlation with the abundance of *Cx. pipiens molestus* (β = −0.149, *p* < 0.001) and no correlation with that of *Cx. quinquefasciatus* (β = −0.022, *p* = 0.445). For RH, positive correlations were observed with *Ae. albopictus* (β = 0.310, *p* < 0.001), whereas no correlation was found between RH and the abundance of *Cx. quinquefasciatus* (β < 0.001, *p* = 0.99), *Cx. pipiens molestus* (β = −0.044, *p* = 0.202), and *Cx. tritaeniorhynchus* (β = 0.1, *p* = 0.08).

### 3.4. Spatial Difference in Mosquito Diversity and Abundance

Mosquito diversity varied notably among the study sites. Both Simpson and Shannon’s diversity indices (1 − *D* and *H*′) were highest at ZOO (1 − *D* = 0.78; *H*′ = 2.02), followed by TAS (1 − *D* = 0.68; *H*′ = 1.34), and lowest at CMDS (1 − *D* = 0.58; *H*′ = 1.28). The lower indices at CMDS and TAS reflect a community structure characterized by stronger dominance of a few species.

**Figure 4:**
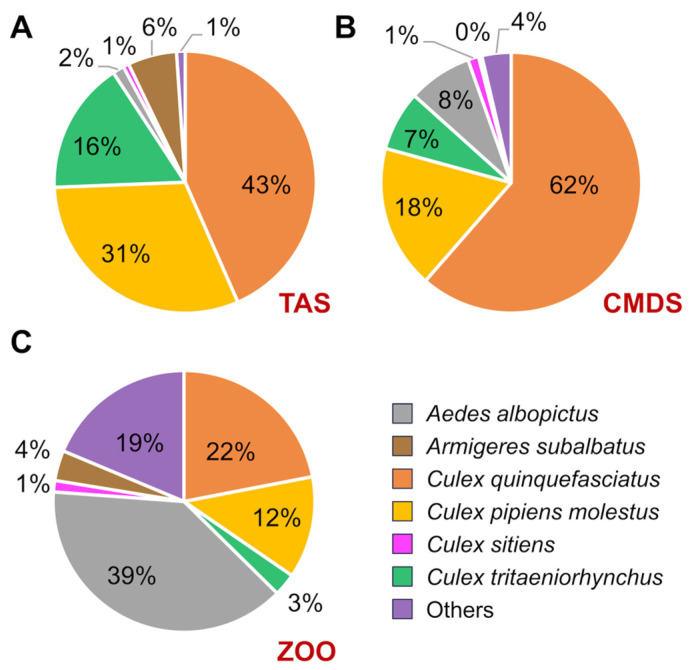
Relative abundances of mosquito species caught at three study sites in Taipei, Taiwan. (**A**) Species abundance at Taipei Animal Shelter (TAS); (**B**) species abundance at Cho Mama Dog Shelter (CMDS); (**C**) species abundance at Taipei Zoo (ZOO).

Among the study sites, the highest number of mosquitoes was recorded at CMDS (*n* = 1431; 74%), followed by TAS (*n* = 281; 15%) and ZOO (*n* = 214; 11%). The relative abundances of mosquito species caught at each site are presented in Figure 4. Six species (*Cx. quinquefasciatus*, *Cx. pipiens molestus*, *Cx. sitiens*, *Cx. tritaeniorhynchus*, *Ae. albopictus*, and *Ar. subalbatus*) were caught at all sites. *Culex quinquefasciatus* was the most abundant species at CMDS and TAS, followed by *Cx. pipiens molestus* and *Ae. albopictus*. *Aedes albopictus* was the most frequently caught species at ZOO, followed by *Cx. quinquefasciatus* and *Cx. pipiens molestus*.

## 4. Discussion

We investigated the seasonal dynamics of mosquito populations in Taipei, Taiwan. Mosquitoes were present year-round, with population troughs recorded from September to November in 2023 and October to December in 2024. The delayed trough in 2024 likely resulted from higher average monthly temperatures compared to 2023. *Culex quinquefasciatus*, *Cx. pipiens molestus*, *Ae. albopictus*, and *Cx. tritaeniorhynchus* accounted for over 90% of the collected specimens, each exhibiting distinct seasonal patterns.

*Culex quinquefasciatus* was the most prevalent species throughout the two-year study period, exhibiting a consistent year-round presence. This is supported by our GLMM analysis, which revealed that temperature did not significantly influence the abundance of this species. However, its prevalence reached relative lows in September–November 2023 and July–December 2024, a pattern consistent with prior findings in Taipei [20]. It is typically found in peridomestic habitats, including artificial containers, catch basins, and wastewater effluent [23]. The 2023 population trough (September–November) may be attributed to three consecutive typhoons in August, September, and October, as heavy rainfall likely flushed out its breeding sites. Similarly, the 2024 population trough (July–December) may be attributed to heavy rainfall in June, July, and August, followed by two typhoons in September and October, suggesting that typhoons and torrential rain are the key factors responsible for the low population level of this species. Notably, *Cx. quinquefasciatus* is an established vector of several pathogens of medical and veterinary importance, including the filarial nematode *Wuchereria bancrofti*, the canine heartworm *Dirofilaria immitis*, and various arboviruses, such as West Nile virus (WNV) and St. Louis encephalitis virus [24,25]. Of the resulting diseases, only canine heartworm disease is currently prevalent in Taiwan [26]. High mosquito density throughout the year poses a risk to dogs’ health.

The second most abundant species was *Cx. pipiens molestus*, an invasive species first reported in Taipei in 1996 [27]. In this study, its abundance peaked from December to April, a period corresponding to the cooler months in Taipei. This cold-tolerance feature is further supported by our GLMM analysis, which revealed a significant negative correlation between temperature and the abundance of this species. Our findings are consistent with previous research in Taipei, which identified *Cx. pipiens molestus* as the only active mosquito species during January and February [28]. Several unique biological traits of *Cx. pipiens molestus*, including autogeny, hypogeny, stenogamy, and absence of diapause, distinguishes it from other members of the *Cx. pipiens* complex [29]. These characteristics enable this species to remain well-adapted and active during winter [28,29,30,31]. Remarkably, *Cx. pipiens molestus* primarily feeds on mammals, which distinguishes it from the bird-feeding *Cx. pipiens pipiens* [31]. Consequently, it has a higher potential to transmit pathogens to humans. It has been identified as the primary vector of *W. bancrofti*, a human-specific filarial nematode responsible for lymphatic filariasis in Egypt [32]. It has also been implicated in the transmission of WNV and other arboviruses, such as Japanese encephalitis virus (JEV) and Usutu virus, under laboratory conditions [33,34,35].

*Aedes albopictus* ranked third, with collections concentrated between June and October; this seasonal trend is similar to that reported in a previous large-scale survey in Taiwan [16] and a regional study conducted in Kaohsiung [18]. This observed seasonal preference is further supported by our GLMM analysis, which revealed that temperature and RH were positively correlated with the abundance of this species, confirming its affinity for warmer and humid conditions. This species breeds in natural water pools, such as those found in tree holes, bamboo internodes, and bromeliads; however, it has also adapted to suburban and urban settings and can breed in artificial containers, such as discarded tires and water storage vessels [36]. Moreover, given its opportunistic feeding behavior [37], *Ae. albopictus* acts as a vector for numerous viral pathogens that infect both animals and humans. For example, it can transmit DENV, CHIKV, ZIKV, WNV, and several other arboviruses [38]. Of the resulting diseases, only dengue fever is often caused by imported cases with subsequent local community transmission in Taiwan. Most outbreak cases have been reported to occur between June and November in Taiwan [39] and are closely associated with the seasonal dynamics of the mosquito vectors.

*Culex tritaeniorhynchus* ranked fourth, with its abundance peaking in August and September; this trend corresponds to findings of a previous study conducted in northern Taiwan [14] but is later than the July–August peak reported in Taipei from 1960 to 1963 [20]. This indicates that urban development and changes in land use can induce shifts in the occurrence and seasonal patterns of this mosquito species. This species predominantly breeds in rice fields [14,40], and its population dynamics are therefore closely linked to the rice-growing cycle [41]. This seasonal activity is further supported by our GLMM analysis, which revealed a significant positive correlation between temperature and the abundance of this species, confirming its affinity for warmer conditions. Additional breeding habitats include ground pools, streams, swamps, shallow marshes, irrigation ditches, and animal hoof prints [24]. However, none of the three study sites in this study is situated near rice fields. Lack of breeding habitats may have contributed to the relatively low abundance of *Cx. tritaeniorhynchus* observed in this study. Furthermore, this species commonly feeds on pigs and cattle, mainly exhibits nocturnal activity, and primarily feeds outdoors [24,40]. *Culex tritaeniorhynchus* is the primary vector of JEV [40,41,42]. Japanese encephalitis remains endemic in Taiwan. In a large-scale surveillance study conducted from 2005 to 2012, *Cx. tritaeniorhynchus* was the most common mosquito species detected in pig farms and wetlands and was identified as the major vector responsible for the transmission of JEV in Taiwan [42]. In addition to JEV, field-caught *Cx. tritaeniorhynchus* has been found to be infected with other human and animal viruses, including ZIKV, CHIKV [43], and Tembusu virus [44].

Beyond the abovementioned dominant species, 27 additional species across eight genera, including *Anopheles*, *Armigeres*, *Culex*, *Malaya*, *Mansonia*, *Mimomyia*, *Ochlerotatus*, and *Uranotaenia*, were collected in this study. *Armigeres subalbatus*, *Cx. mimulus*, and *Cx. sitiens* were the fifth, sixth, and seventh most abundant species in this study, respectively. However, their sample sizes were less than 32 during the two-year study period; these sample sizes are too small for meaningful seasonal analyses.

Mosquito community structure varied notably among the study sites. While both Simpson and Shannon’s diversity indices were highest at ZOO, followed by TAS, and lowest at CMDS, the overall mosquito abundance at CMDS exceeded that recorded at ZOO. These results indicate that the CMDS mosquito community was characterized by the strong dominance of a few species, whereas the ZOO site hosted a more heterogeneous and balanced assemblage. *Culex quinquefasciatus* was the most abundant species at both CMDS and TAS. This species is generally associated with peridomestic habitats, such as artificial containers, catch basins, and wastewater effluent [23], which are abundant in the urban residential areas near these two sites. Furthermore, *Cx. pipiens molestus*, known for breeding in underground water storage and septic tanks in Taiwan [28], was the second most abundant species at both CMDS and TAS. In contrast, while *Ae. albopictus* ranked third in overall abundance; it was the most frequently caught species at ZOO. The diverse environment at ZOO provides a wide range of oviposition sites, allowing this species to exploit both artificial and natural water sources for breeding.

A potential limitation of this study is the reliance on CO_2_-baited CDC light traps, which are primarily designed to capture crepuscular and nocturnal mosquitoes. This methodology likely leads to an underestimation of diurnal species across the study sites. For instance, although *Ae. albopictus* was the third most prevalent species in our two-year survey, its actual abundance is likely higher given its predominantly diurnal activity pattern [36]. Similarly, because members of the genera *Ochlerotatus* and *Malaya* are generally diurnal [45,46,47], their populations may have been underrepresented. Furthermore, trap selection significantly influences the mosquito composition captured; previous studies have reported that *Ochlerotatus japonicus* is more effectively collected using gravid traps rather than CO_2_-baited light traps [48]. In this study, *Oc. japonicus shintienensis* was rarely detected (only two individuals at the ZOO site), a finding that may be attributed to trap type rather than low local density. To achieve a more taxonomically comprehensive assessment in future surveillance, incorporating diverse trapping methods, such as BG-Sentinel traps, gravid traps, and larval surveys, would be essential to mitigate the inherent biases associated with any single collection method [24].

Our findings provide critical insights into the epidemiology of mosquito-borne diseases and the complex relationships between vectors and their environments, which are essential for informing effective vector control strategies. Specifically, by identifying species-specific seasonal peaks, this study offers an empirical basis for synchronizing control interventions, such as source reduction and larviciding, with periods of maximum vector activity. Furthermore, the observed spatiotemporal variability in mosquito abundance supports the development of site-specific surveillance protocols rather than a one-size-fits-all approach. Beyond practical control, our results also support a One Health perspective by highlighting the intricate dependencies among environmental conditions, mosquito ecology, and the risk of pathogen transmission at the human–animal–environment interface. The presence of bridge vectors capable of transmitting zoonotic pathogens underscores the link between animal reservoirs and potential human exposure. Collectively, these findings emphasize that sustainable disease prevention requires an integrated strategy that combines environmental management, animal health surveillance, and human health protection, thereby aligning with One Health principles to reduce the overall risk of mosquito-borne diseases in metropolitan environments.

## 5. Conclusions

In this study, mosquito surveillance in Taipei revealed a community structure strongly dominated by four species. Mosquitoes were present year-round. Species-specific temporal dynamics reflected differences in ecological adaptation. Furthermore, spatial heterogeneity in both mosquito abundance and species composition was noted among the study sites, underscoring the influence of local environmental conditions within an urban landscape. Understanding the seasonal dynamics of mosquito species is crucial, as it can provide valuable insights into the epidemiology of mosquito-borne pathogens and the complex relationships between vectors and their environments. These findings can help inform effective, targeted vector control strategies for reducing disease transmission. Moreover, this research supports One Health perspectives by highlighting the connections among human, animal, and environmental health for shaping mosquito ecology and reducing disease risk.

## Figures and Tables

**Table 1:** Female mosquitoes caught at three study sites in Taipei, Taiwan.

Mosquito Species	Subfamily	CMDS ^1^	TAS ^2^	ZOO ^3^	Total
*Anopheles ludlowae*	Anophelinae	0	0	1	1
*Anopheles sinensis*	Anophelinae	5	2	0	7
*Anopheles tessellatus*	Anophelinae	0	0	1	1
*Aedes albopictus*	Culicinae	114	4	83	201
*Armigeres baisasi*	Culicinae	1	0	2	3
*Armigeres omissus*	Culicinae	0	0	1	1
*Armigeres subalbatus*	Culicinae	6	17	8	31
*Culex annulus*	Culicinae	1	0	1	2
*Culex bicornutus*	Culicinae	0	0	1	1
*Culex bitaeniorhynchus*	Culicinae	0	0	6	6
*Culex infantulus*	Culicinae	0	0	1	1
*Culex macrostylus*	Culicinae	3	0	0	3
*Culex mimulus*	Culicinae	16	0	9	25
*Culex nigropunctatus*	Culicinae	3	0	0	3
*Culex pallidothorax*	Culicinae	8	0	7	15
*Culex pipiens molestus*	Culicinae	255	87	27	369
*Culex quinquefasciatus*	Culicinae	879	122	47	1048
*Culex sitiens*	Culicinae	19	2	3	24
*Culex tritaeniorhynchus*	Culicinae	106	46	6	158
*Malaya genurostris*	Culicinae	7	0	0	7
*Mansonia uniformis*	Culicinae	1	0	0	1
*Mimomyia fusca*	Culicinae	0	0	1	1
*Mimomyia luzonensis*	Culicinae	0	0	1	1
*Ochlerotatus dorsalis*	Culicinae	1	0	0	1
*Ochlerotatus elsiae vicarious*	Culicinae	0	1	0	1
*Ochlerotatus japonicus shintienensis*	Culicinae	0	0	2	2
*Uranotaenia annandalei*	Culicinae	2	0	1	3
*Uranotaenia macferlanei*	Culicinae	0	0	2	2
*Uranotaenia nivipleura*	Culicinae	0	0	1	1
*Uranotaenia novobscura*	Culicinae	3	0	2	5
*Uranotaenia yaeyamana*	Culicinae	1	0	0	1
Total		1431	281	214	1926

^1^ CMDS: Cho Mama Dog Shelter, Neihu district, Taipei. ^2^ TAS: Taipei Animal Shelter, Neihu district, Taipei. ^3^ ZOO: Taipei Zoo, Wenshan district, Taipei.

## Data Availability

The original contributions presented in this study are included in the article/Supplementary Material. Further inquiries can be directed to the corresponding author.

## Supplementary Materials

The following supporting information can be downloaded at: https://www.mdpi.com/article/10.3390/insects17060592/s1. Table S1: Summary of monthly average temperature, rainfall, and relative humidity (RH) in Neihu and Wenshan districts of Taipei, Taiwan.
