# Supplementary material for: Seasonal Population Dynamics of Mosquitoes in Taipei, Taiwan
Source: Insects. 2026 Jun 5;17(6):592. doi: 10.3390/insects17060592 (PMC13299400; doi:10.3390/insects17060592)
Supplement: Supplementary file 1 [file insects-17-00592-s001.zip › insects-4204886-supplementary.pdf]

Table S1. Summary of monthly average temperature, rainfall, and relative humidity (RH) in Neihu and Wenshan districts of Taipei, Taiwan.

**District**		**Jun 2023**	**Jul 2023**	**Aug 2023**	**Sep 2023**	**Oct 2023**	**Nov 2023**	**Dec 2023**	**Jan 2024**	**Feb 2024**	**Mar 2024**	**Apr 2024**	**May 2024**
Neihu	Temperature (°C)	29 ± 1.8	30.9 ± 1.4	30.1 ± 1.4	29.3 ± 1.0	25.2 ± 1.7	22.4 ± 3.2	18.6 ± 3.6	16.9 ± 3.2	18 ± 3.6	19.2 ± 4.0	24.8 ± 2.6	25.2 ± 2.4
	Rainfall (mm)	393	136.5	339.5	105	110.5	13.5	86.5	29	35	111	203.5	114
	RH (%)	75 ± 6.2	68 ± 7.9	74 ± 7.5	69 ± 5.3	70 ± 7.2	67 ± 6.3	74 ± 7.6	69 ± 8.3	73 ± 8.8	71 ± 10.5	76 ± 6.4	73 ± 9.5
Wenshan	Temperature (°C)	27.6 ± 1.5	29.3 ± 1.1	28.5 ± 1.4	27.8 ± 1.1	24.3 ± 1.7	21.2 ± 3.1	17.8 ± 3.3	15.8 ± 3.1	16.9 ± 3.3	18.2 ± 3.9	23.9 ± 2.3	24.3 ± 2.2
	Rainfall (mm)	358	139	439	121.5	150.5	49.5	112	93	66	126	201	186
	RH (%)	82 ± 5.8	75 ± 5.9	80 ± 6.9	77 ± 5.3	80 ± 7.4	77 ± 7.4	84 ± 7.1	79 ± 8.3	82 ± 8.5	78 ± 10.4	83 ± 5.2	80 ± 8.0

**District**		**Jun 2024**	**Jul 2024**	**Aug 2024**	**Sep 2024**	**Oct 2024**	**Nov 2024**	**Dec 2024**	**Jan 2025**	**Feb 2025**	**Mar 2025**	**Apr 2025**	**May 2025**
Neihu	Temperature (°C)	28.5 ± 2.7	30.4 ± 1.5	29.7 ± 1.2	29.1 ± 2.0	25.5 ± 2.1	21.8 ± 3.0	17.4 ± 2.6	16 ± 2.4	16 ± 2.7	18.6 ± 3.9	22.5 ± 3.5	26.5 ± 2.9
	Rainfall (mm)	325	320	236.5	511.5	440	66	23	36.5	82.5	213.5	187	343.5
	RH (%)	76 ± 7.6	72 ± 6.5	74 ± 5.4	74 ± 8.1	74 ± 6.3	72 ± 7.1	69 ± 6.4	65 ± 9.2	72 ± 8.7	71 ± 12.3	71 ± 11.1	73 ± 8.1
Wenshan	Temperature (°C)	27.4 ± 2.2	28.9 ± 1.1	28.5 ± 1.0	28.3 ± 1.7	25.2 ± 2.1	21.3 ± 3.2	16.7 ± 2.5	15.2 ± 2.5	15.6 ± 2.7	17.7 ± 3.7	21.7 ± 3.2	24.8 ± 2.5
	Rainfall (mm)	384.5	397	176	478.5	525	98.5	38.5	81	130	272.5	193	394.5
	RH (%)	83 ± 5.8	79 ± 5.9	81 ± 4.6	79 ± 9.3	83 ± 6.9	82 ± 7.8	79 ± 7.8	73 ± 10.0	80 ± 8.3	79 ± 10.3	78 ± 9.7	79 ± 7.5

1. Meteorological data were obtained from the Climate Observation Data Inquiry System (CODIS) of the Central Weather Administration, Taiwan.
2. Data are expressed as mean ± standard deviation (SD).
3. RH data for the Neihu district were not available; therefore, data from the adjacent Shilin district were used as a proxy.
